# Cholesteryl Ester Promotes Mammary Tumor Growth in MMTV-PyMT Mice and Activates Akt-mTOR Pathway in Tumor Cells

**DOI:** 10.3390/biom11060853

**Published:** 2021-06-08

**Authors:** Lengyun Wei, Xuyang Lu, Shengmei Weng, Shenglong Zhu, Yongquan Chen

**Affiliations:** 1 Wuxi School of Medicine, Jiangnan University, Wuxi 214122, China; 7150112026@vip.jiangnan.edu.cn (L.W.); 6170112151@stu.jiangnan.edu.cn (X.L.); 6192805007@stu.jiangnan.edu.cn (S.W.); shenglongzhu@jiangnan.edu.cn (S.Z.); 2 Wuxi Translational Medicine Research Center and Jiangsu Translational Medicine Research Institute Wuxi Branch, Wuxi 214122, China; 3 School of Food Science and Technology, Jiangnan University, Wuxi 214122, China

**Keywords:** breast cancer, cholesteryl ester, SOAT1, avasimibe

## Abstract

The association between intratumoral cholesteryl ester (CE) and tumor progression has been reported previously. The objective of our study was to investigate a causal effect of CE on mammary tumor progression. Using MMTV-PyMT (MMTV-polyoma virus middle T) transgenic mice and breast tumor cell MCF-7, we show that both exogenous and endogenous CE can increase mammary tumor growth, that CE upregulates the AKT/mTOR pathway, and that CE synthesis blockade suppresses this signaling pathway. Our data suggest that SOAT1, a sterol O-acyltransferase, may be a potential target for the treatment of breast cancer.

## 1. Introduction

Cholesterol esterification is a mechanism the body uses to store and transfer cholesterol and to avoid cellular toxicity of the excess of unesterified cholesterol [1,2]. However, the inert condition of CEs is dramatically changed if cholesterol is esterified to a polyunsaturated fatty acid or subjected to a higher degree of enrichment [2,3]. In recent years, mounting evidence has indicated that high expression of sterol O-acyltransferase 1 (SOAT1) is accompanied by high CE content in glioblastoma, pancreatic cancer, prostate cancer, and some other tumors [4,5,6,7]. Some studies have demonstrated that intratumoral CE accumulation is intimately linked to the proliferation and aggressive potential of breast cancer and that the migration of MDA-MB-231 breast cancer cells depends on the availability of exogenous lipids and cholesterol esterification [3,8]. Although the abnormal accumulation of CEs may pose a threat to women’s health [9], few studies have investigated the causal effect of CE on breast cancer to date.

The enzymes that catalyze the formation of CE from cholesterol and long-chain fatty acids in cells are sterol O-acyltransferases (SOAT1/2) [10,11]. SOAT1 (also known as ACAT1) is widely expressed in many different tissues, whereas SOAT2 (also known as ACAT2) is restricted to hepatic and gastrointestinal tissues [12]. Inhibition of cholesterol esterification via blocking SOAT1 has been reported to significantly reduce the proliferation of some solid tumor cells [13,14]. The potential role of CE or SOAT1 in breast cancer remains poorly understood. Therefore, we investigated the effect of exogenous and endogenous CE, using diet and SOAT1 inhibitor avasimibe, on tumor growth in a mouse mammary tumor model. Furthermore, a possible signaling pathway responsible for breast cancer cell growth was also investigated.

## 2. Materials and Methods

### 2.1. MMTV-PyMT Mice and Diets

FVB/N Tg (MMTV-PyMT) (hereafter referred to as PyMT) mice were used in these studies. In this polyoma virus middle T (PyMT) breast cancer mouse model, the PyMT oncogene is expressed in the mammary epithelium under the control of mouse mammary tumor virus (MMTV)-long terminal repeat promoter element [15]. When expressed, the middle T protein is inserted into the cell membrane and undergoes phosphorylation by kinases of the Src family, thereby activating oncogenic cell signaling [16]. This mouse model displays widespread transformation of the mammary gland and has been utilized extensively as a preclinical mouse model. All in vivo experimental procedures were approved by the Animal Care Research Committee of Jiangnan University. Mice were maintained in a temperature-controlled facility with a 12 h light–dark cycle and ad libitum access to water and diet. Chow (CW), chow + 0.5%CE (0.5CE), chow + 1%CE (1CE), fat diet (FD) with 30% of energy derived from fat, cholesterol diet (CD) with 2% cholesterol in chow, and fat–cholesterol diet (FCD) with 2% cholesterol in FD were produced commercially by Trophic Animal Feed High-Tech Co., Ltd. (Nantong, China). In spontaneous tumor formation experiments, mammary glands were palpated every other day to monitor tumor latency and progression. Tumors were dissected, weighed, embedded into paraffin, and cut into 5-micron-thick sections. Immunohistochemical staining was performed to detect the expression of Ki-67, as described previously [17].

### 2.2. Drugs and Reagents

Avasimibe was purchased from Med Chem Express (Shanghai, China), stored at 30 mg/mL in −80 °C, and diluted at a 1:10 ratio with phosphate buffer saline (PBS) when used. CE was purchased from Tokyo Chemical Industry (Shanghai, China).

### 2.3. Clinical Data Collection and Analysis

The clinical-pathological features of patients and tumor samples are summarized in Table 1. Tumor tissue samples were obtained from mastectomy or lumpectomy specimens which were frozen in liquid nitrogen and stored at the tissue core facility of the second affiliated hospital of Chong Qing. Sixteen tumor samples were selected retrospectively and the Ki-67 index was evaluated by immunohistochemistry.

### 2.4. Lipidomics and CE Analysis

Lipids were extracted from serum and measured by liquid chromatography/mass spectrometry (LC/MS), and CE was extracted and analyzed by gas chromatography/mass spectrometry (GC/MS), as described previously [18,19]. Relative level of CE was normalized to tissue weight.

### 2.5. Cell Culture

MCF-7 (Institute of Cell Biology, Shanghai, China) was cultured in Dulbecco’s modification of Eagle’s medium (DMEM, C11960500BT, Gibco, Shanghai, China) supplemented with 10% (*v*/*v*) fetal bovine serum (FBS, 10099-141; Gibco) and 1% (*v*/*v*) penicillin/streptomycin at 37 °C and 5% CO_2_ in a humidified chamber. The medium was renewed every 2–3 days.

### 2.6. Cell Counting

Cells were cultured in 6-well plates at a density of 100,000 cells per well in 2 mL medium and incubated for 24 h. When grown to 60–70% confluency, cells were treated with various concentrations of avasimibe (0.001, 0.01, 0.1, 1, or 10 μM) and incubated for 48 h. Cells were then digested by trypsin and counted using a hemacytometer.

### 2.7. Flow Cytometry Analysis of Cell Cycle

Cell cycle was determined with propidium iodide (Sigma-Aldrich, St. Louis, MO, USA) staining method. Cells were treated for 48 h, collected, fixed in 70% ethanol, and stored at 4 °C prior to cell cycle analysis. After the removal of ethanol by centrifugation, cells were washed with PBS twice, stained with a solution containing 100 μg/mL RNase A, 0.2% Triton X-100, and 50 μg/mL propidium iodide, and analyzed on the BD Accuri™ C6 Plus Flow Cytometer (BD Biosciences, Franklin Lakes, NJ, USA) with Novo Express Software (ACEA Biosciences Inc., Beijing, China)

### 2.8. Western Blot

Whole cells were lysed by lysis buffer (RIPA buffer contains protease inhibitors and phosphatase inhibitors). Protein concentration was measured using the Pierce BCA Protein Assay Kit (Thermo Scientific, Shanghai, China). A total of 30 μg of protein was separated on SDS-PAGE gels (mTOR/P-mTOR 6% gel, pAKT473/AKT/β-Actin 12% gel, pp70s6k/p70s6k 10% gel) and transferred onto Immobilon-P membrane (IPVH07850; Millipore, Darmstadt, Germany). Membranes were first blocked with 5% non-fat milk in Tris-buffered saline with 1% Tween 20 (TBST) at room temperature for 1 h, then incubated overnight at 4 °C with the following primary antibodies: pAKT473 (Cell Signaling Technology, Boston, MA, USA), pAKT308 (Cell Signaling Technology, Boston, MA, USA), AKT (Cell Signaling Technology, Boston, MA, USA), pmTOR (Cell Signaling Technology, Boston, MA, USA), mTOR (Cell Signaling Technology, Boston, MA, USA), pp70s6k (Cell Signaling Technology, Boston, MA, USA), and p70s6k (Cell Signaling Technology, Boston, MA, USA), diluted at a 1:1000 ratio in TBST with 3% bovine serum albumin (BSA), washed with TBST, and finally incubated with HRP-labelled goat anti-mouse or anti-rabbit IgG secondary antibodies at room temperature for 1.5 h. The proteins were visualized by Plus-enhanced chemiluminescence using Universal Hood III (Bio-Rad, Hercules, CA, USA).

### 2.9. Statistical Analysis

Data were analyzed with Prism 8.0 (Graphpad software, San Diego, CA, USA). Significance was determined using an unpaired two-tailed *t* test for single-variable experiments. Then, Tukey’s one-way analysis of variance (ANOVA) was used for multiple comparisons. *p* < 0.05 was considered significant. When using letters to show statistical differences, there was no significant difference between groups marked with the same letter and there were significant differences between any two groups marked with different letters. Simca was used to generate a principal-component analysis (PCA) based on taxonomy or lipid matrix.

## 3. Results

### 3.1. Intratumor Cholesteryl Ester Level Is Elevated in Tumor Tissues

**Figure 1:**
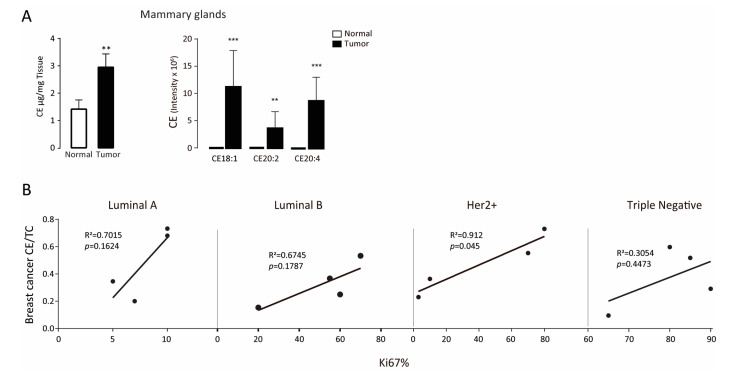
Cholesteryl ester level is elevated in tumor tissues. (**A**) Cholesteryl ester levels in mammary glands isolated from wild-type (WT) and PyMT mice. Error bars represent standard error of the mean (SEM). ** *p* < 0.01, *** *p* < 0.001, as determined using a two-tailed Student’s *t*-test. (**B**) Correlation between intratumoral CE and Ki-67 positive population in different breast carcinoma subtypes. The correlation was statistically significant (*p* < 0.05) in Her2+ subtype but not in Luminal A, Luminal B, and Triple Negative subtypes.

Mammary gland and tumor tissues from wild-type and PyMT female mice at age 12 weeks were collected for CE content measurement via GC-MS. Results indicated that levels of CE, especially CE18:1 and CE20:4, were significantly increased in the mammary glands of PyMT mice compared with those of age-matched wild-type mice (Figure 1A).

To explore the correlation between CE levels and tumor malignancy, we selected 16 clinical tumor samples from the second affiliated hospital of Chong Qing (detailed pathological characteristics are shown in Table 1) and analyzed intratumor CE content. We found that the intratumoral CE level was correlated with Ki-67 positivity in four subtypes of breast carcinoma, which reached statistical significance (*p* = 0.045) in Her2^+^ carcinoma (Figure 1B).

### 3.2. Exogenous Cholesteryl Ester Decreases Tumor-Free Survival

To assess the role of exogenous CE on mammary tumor development and progression, we divided 4-week-old PyMT mice into three groups (*n* = 6 per group) and fed them with chow diet, chow plus 0.5% CE, or 1% CE, respectively. Mice were examined by palpation for tumor formation in the cohort every 2 days and were sacrificed at the age of 12 weeks.

**Figure 2:**
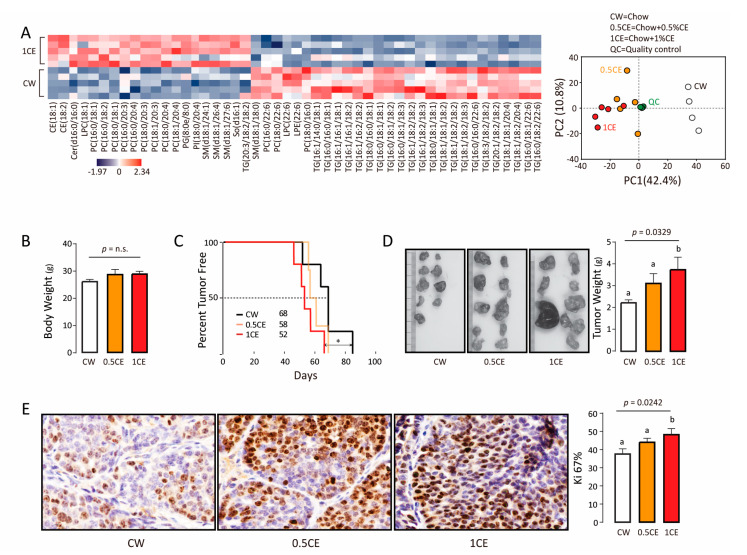
Dietary cholesteryl ester decreases tumor free survival. (**A**) Left: Heat maps of differentially expressed lipids in the serum of mice received diet with or without cholesteryl ester. CE, cholesterol ester; Cer, ceramides; LPC, lysophosphatidyl choline; PC, phosphatidyl choline; PG, phosphatidyl glycerol; PI, phosphatidyl inositol; SM, sphingomyelin; So, sphingosine; TG, triglyceride; LPE, lysophosphatidyl ethanolamine. 8:0, caprylic acid; 14:0, myristic acid; 16:0, palmitic acid; 16:1, palmitoleic acid; 18:0, stearic acid; 18:1, oleic acid; 18:2, linoleic acid; 18:3, α-Linolenic acid; 20:1, gondoic acid; 20:3, dihomo-γ-linolenic acid; 20:4, arachidonic acid; 22:6, docosahexaenoic acid; 24:1, nervonic acid; 26:4, hexacosatetraenoic acid; 27:6, heptacosahexaenoic acid. Right: Principal-component analysis (PCA) of the matrix data. PyMT mice received an increasing dose cholesteryl ester diet (with 5, 10 mg cholesteryl ester per kg added, *n* = 5). (**B**) Body weight of each group. (**C**) Tumor latency of each group. * *p* < 0.05. (**D**) Tumor weight of each group. Values (means ± S.E.M.) with different letters indicate statistical significance, *p* < 0.05. (**E**) Immunohistochemical staining of Ki-67 in mammary gland (tumor). Values (means ± S.E.M.) with different letters indicate statistical significance, *p* < 0.05.

CE diet significantly altered serum lipid profile (Figure 2A) without affecting body weight (Figure 2B). Tumor latency was significantly shortened in 1% CE diet group compared to the chow group (50% tumor onset in 52 days vs. 68 days) (Figure 2C). Total tumor weight per mice (Figure 2D) and tumor cell proliferation as indicated by Ki-67 positivity (Figure 2E) increased with rising amounts of CE. Thus, exogenous CE accelerates tumor onset and progression.

### 3.3. Endogenous Cholesteryl Ester Promotes Tumor Growth

In order to explore the role of endogenous CE in breast cancer, we divided 4-week-old PyMT mice into five groups (*n* = 6 per group). Mice were fed with chow diet (CW), fat diet (30% fat energy, FD), cholesterol diet (CW + 2% cholesterol, CD), fat–cholesterol diet (FD + 2% cholesterol, FCD), or fat–cholesterol diet with SOAT1 inhibitor avasimibe (gaveged 12 mg/kg/d) (FCD + avasimibe, FCI).

**Figure 3:**
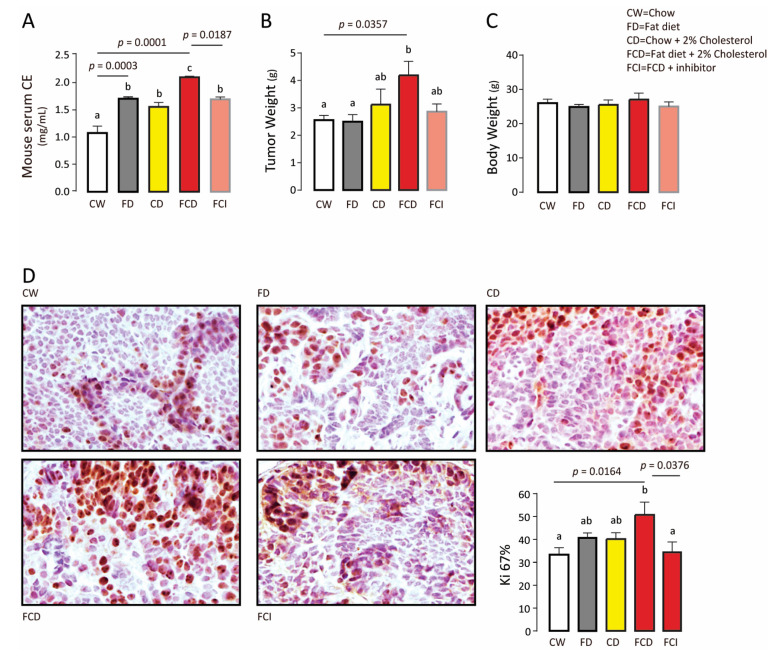
Endogenous cholesteryl ester promotes tumor proliferation. (**A**) Serum CE of each group. (**B**) Tumor weight of each group. (**C**) Body weight of each group. (**D**) Immunohistochemical staining of Ki-67 in mammary gland (tumor). For experiments where necessary, ANOVA (Tukey’s test) was performed and *p* < 0.05 was considered as significant. Values (means ± S.E.M.) with different letters indicate statistical significance.

Serum CE level (Figure 3A) and tumor weight (Figure 3B) increased significantly in the FCD group compared to the FD and CD groups without altering body weight (Figure 3C). Inhibition of SOAT1 using avasimibe reduced serum CE level and tumor weight (Figure 3A,B) as well as Ki-67 positive cell population (Figure 3D). Therefore, inhibition of endogenous CE synthesis reduces mammary tumor growth.

### 3.4. CE Synthase Inhibitor Reduces AKT and mTOR Activation and Suppresses Tumor Cell Proliferation

**Figure 4:**
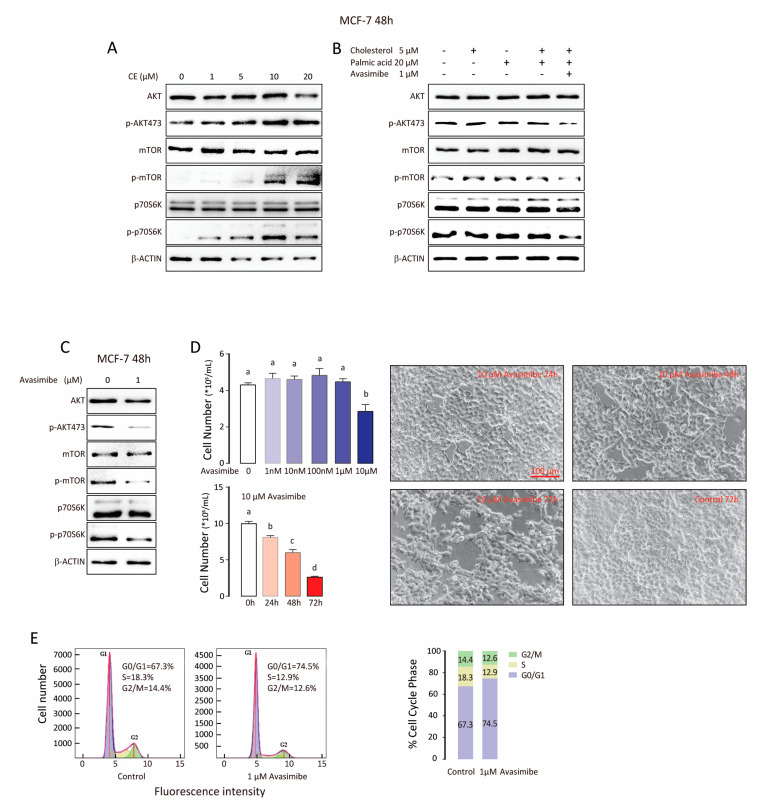
CE synthase inhibitor reduces AKT and mTOR activation and suppress tumor cell growth. (**A**) Expression of p-AKT, total AKT, p-mTOR, total mTOR, p-p70S6K, and total p-70S6K proteins in MCF-7 cells treated with cholesteryl ester. β-Actin was used as loading control. (**B**) Expression of p-AKT, total AKT, p-mTOR, total mTOR, p-p70S6K and total p-70S6K proteins in MCF-7 cells treated with cholesterol or palmic acid alone or in combination with or without SOAT1 inhibitor avasimibe. (**C**) Expression of p-AKT, total AKT, p-mTOR, total mTOR, p-p70S6K, and total p-70S6K proteins in MCF-7 cells treated with 1 μM avasimibe. β-Actin was used as loading control. (**D**) Dose/time dependent cell proliferation after treatment with or without avasimibe. (**E**) Cell cycle analysis after treatment with or without avasimibe. For experiments where necessary, ANOVA (Tukey’s test) was performed and *p* < 0.05 was considered as significant. Values (means ± S.E.M.) with different letters indicate statistical significance.

Previous research has suggested that CE level is up-regulated by PI3K/AKT [4], a pathway important in cell proliferation, survival, and metastasis [20,21,22]. We tested whether the AKT signaling pathway was associated with CE treatment in MCF-7 cells. Western blot analysis was conducted in MCF-7 cells treated with cholesterol oleate (1, 5, 10, and 20 μM) for 48 h (Figure 4A). The phosphorylation level of AKT, mTOR, and p-70S6K increased considerably following cholesterol oleate treatment, which was not affected by cholesterol or palmitic acid alone or in combination (Figure 4B). However, 1 μM avasimibe decreased the phosphorylation of AKT, p-70S6K, and mTOR protein (Figure 4C and Supplementary Figure S1). We then examined the effects of avasimibe on MCF-7 cell growth. MCF-7 cells were exposed to avasimibe at various doses (1 nM to 10 μM) for 24 h to 72 h (Figure 4D). The number of cells was significantly decreased by avasimibe treatment when compared with the control in a dose/time dependent manner. Cell cycle distribution was also evaluated. After treatment with 1 μM avasimibe, the proportion of cells arrested in G0/G1 phase significantly increased from 67.3% to 74.5%, whereas the percentage of cells in S phase notably decreased from 18.3% to 12.9. This suggests that the proliferation of MCF-7 cell may be hindered by G0/G1 phase cell cycle arrest (Figure 4E). Hence, cholesterol oleate may accelerate breast cancer proliferation via the AKT/mTOR pathway and inhibition of CE synthesis by avasimibe can lead to G0/G1 cell cycle arrest.

## 4. Discussion

Group-wise comparisons of clinical samples demonstrated a close relationship between intratumor cholesteryl ester (CE) accumulation and Ki-67, a well-known marker of tumor cell proliferation, poor patient survival, and higher risk of relapse [23,24]. The trend of correlation was consistent in all breast cancer subtypes, especially in Her2+ subtype (*p* < 0.05). Our result, despite consisting of a limited number of samples, is consistent with previous clinical [3] and experimental studies [25,26]. It is noteworthy that pooled analysis does not show a strong correlation between CE level and Ki-67, as the slope of CE levels and Ki67 varies widely among subtypes of breast cancer, especially the Luminal A subtype. Therefore, intratumoral CE as a clinical marker of breast cancer aggressiveness deserves further investigation.

In addition to the correlational analysis, our investigation provides some evidence of a causal relationship between CE and breast cancer. Exogenous and endogenous CE can increase mammary tumor growth and sterol acyltransferase SOAT1 may be a potential target for the treatment of breast cancer.

SOAT1 was identified as an over-expressed gene in the claudin-low intrinsic subtype of breast cancer [27]. In fact, over-expression of SOAT1 in cancer was seen in multiple analyses across a wide variety of cancer types, including brain, breast, cervical, kidney, head and neck cancer, and leukemia [28,29,30,31], suggesting a more general role for high SOAT1 expression in cancer. SOAT1 inhibitor avasimibe was originally used to treat atherosclerosis [32], but failed due to the lack of effectiveness in reducing plaque size in phase III clinical trials. It will be important to demonstrate that avasimibe can be effective in treating cancer clinically. In addition, SOAT1 and/or SOAT2 inhibitors should be further developed in the future.

The AKT/mTOR pathway is among the most frequently dysregulated pathways in patients with breast cancer [21,33]. Previous study has reported that CE accumulation is induced by PI3K/AKT activation [4]. Interestingly, here we find that CE treatment activates AKT/MTOR/S6K in MCF-7 cells, and avasimibe suppresses tumor cell growth likely by inhibiting this signaling pathway. Therefore, it is possible that the CE and AKT/mTOR pathway forms a positive feedback loop, reinforcing each other in tumor cells. Further studies are warranted to explore this mechanism.

## Figures and Tables

**Table 1:** Clinical and pathological characteristics according to breast carcinoma type.

BCa Type	Patient Number	ER	PR	Her-2	Ki-67	Lymph Node Affected	Tumor Size cm	TNM Stage	Age	Menopause
LB	14176	+	−	+	70	5/17	2.5 × 2.5	T2N2M0	38	
	07648	+	−	++	60	5/28	2.8 × 2 × 2	T2N2M0	65	Menopause
	03074	+	−	++	20	0/18	4 × 4 × 2	T1N0M0	63	Menopause
	07857	+	−	++	50–60	0/13	2.5 × 1.5	T2N0M0	60	Menopause
LA	13673	++	++	−	5–10	0/19	2.5 × 2.5	T2N0M0	42	
	03075	+	+	−	5	0/20	1.5 × 1 × 1	T1N0M0	52	
	09276	++	++	−	10	12/15	3.8 × 2.8 × 2	T2N3M0	44	
	08041	++	++	−	10	0/15	3 × 3	T2N0M0	43	
TN	11713	−	−	−	60–70	0/5	2.5 × 2 × 1	T2N0M0	50	
	08223	−	−	−	80	0/15	1.8 × 1.8	T2N0M0	61	Menopause
	13887	−	−	−	90	0/16	3 × 2 × 1.5	T2N0M0	46	
	02587	−	−	−	85	1/31	2.5 × 1.5 × 1.5	T2N1M0	47	
Her-2	11540	−	−	+++	3	0/6	3 × 2 × 1.5	T2N0M0	66	Menopause
	05831	−	−	++	80	0/25		T2N0M0	66	Menopause
	04004	−	−	++	70	0/9	4.5 × 1 × 1	T2N0M0	49	
	07933	−	−	++	10	4/24	2.5 × 2	T2N2M0	37	

LA, luminal A; LB, luminal B; TN, triple negative; ER, estrogen receptor; PR, progesterone receptor.

## Data Availability

Data supporting the findings of this study are available from the first author (Wei, L.) on request.

## Supplementary Materials

The following are available online at https://www.mdpi.com/article/10.3390/biom11060853/s1, Figure S1: whole figure for western blotting.
