# Supplementary figures and images for: Cholesteryl Ester Promotes Mammary Tumor Growth in MMTV-PyMT Mice and Activates Akt-mTOR Pathway in Tumor Cells
Source: Biomolecules. 2021 Jun 8;11(6):853. doi: 10.3390/biom11060853 (PMC8228430; doi:10.3390/biom11060853)

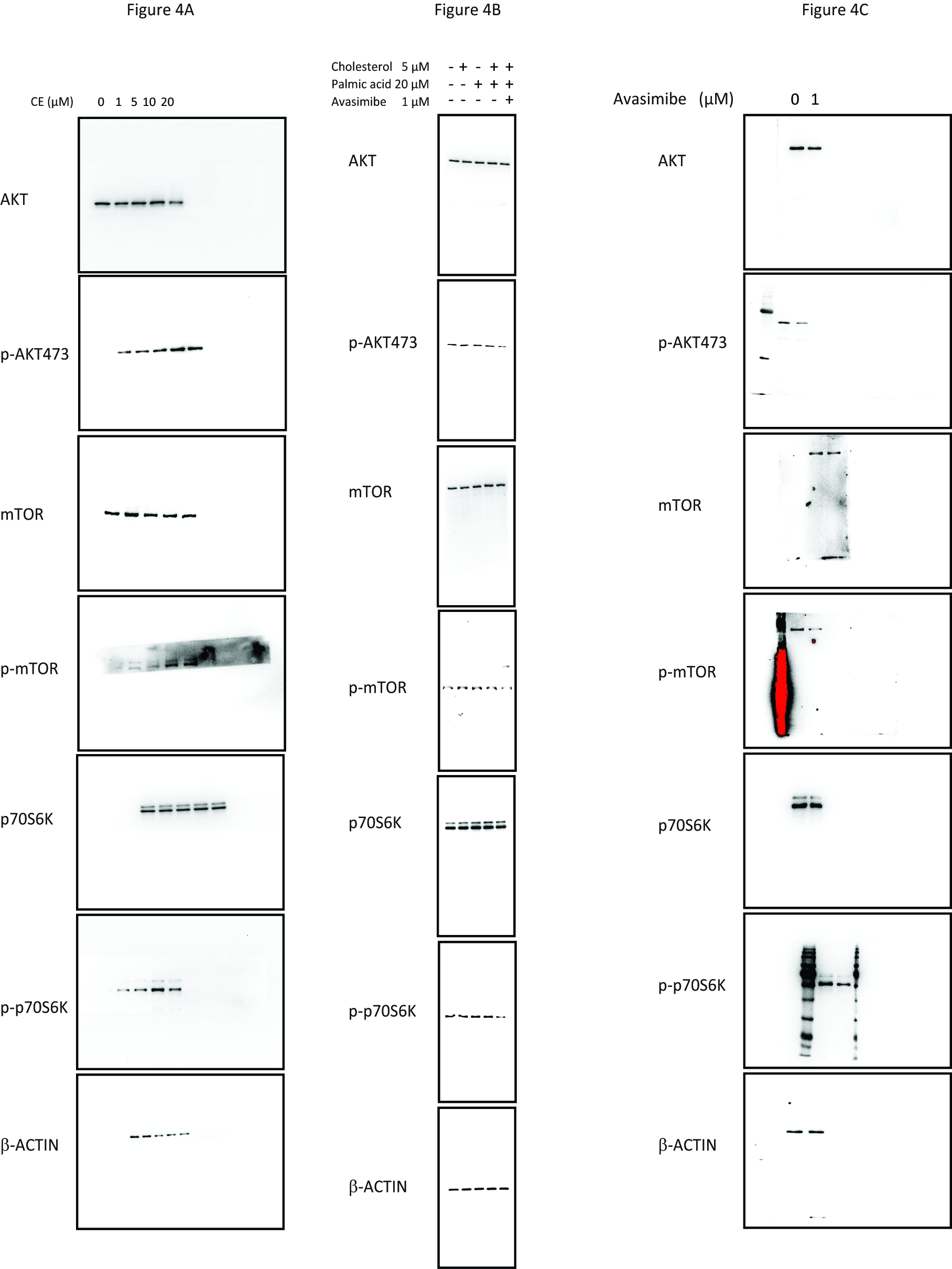

Supplement: Supplementary file 1 [file biomolecules-11-00853-s001.zip › biomolecules-1192601-supplementary.tif]
